# Metabolic Pathways Linking Atherosclerotic Cardiovascular Disease With Metabolic Dysfunction-Associated Steatotic Liver Disease

**DOI:** 10.1007/s11883-026-01425-z

**Published:** 2026-05-13

**Authors:** Koral S. E. Richard, Sumati Rohilla, Reethika Gade, Fabio Arias, Oren Rom

**Affiliations:** 1 Department of Pathology and Translational Pathobiology, Louisiana State University Health Sciences Center-Shreveport, Shreveport, LA 71103 USA; 2 Department of Molecular and Cellular Physiology, Louisiana State University Health Sciences Center-Shreveport, Shreveport, LA 71103 USA

**Keywords:** MASLD, MASH, ASCVD, Amino acid metabolism, Carbohydrate metabolism, Lipid metabolism

## Abstract

**Purpose of Review:**

This review explores the metabolic pathways dysregulated in both atherosclerotic cardiovascular disease (ASCVD) and metabolic dysfunction-associated steatotic liver disease (MASLD), focusing on lipid, carbohydrate, amino acid, and energy metabolism and the specific alterations within major contributing cell types.

**Recent Findings:**

In the setting of metabolic syndrome, lipid and carbohydrate overload impair hepatic metabolism, resulting in the accumulation of lipotoxic species and ensuing cellular damage, inflammation, oxidative stress, and cardiovascular consequences. Amino acid metabolism is emerging as a key regulator of cell fate and function in both MASLD and ASCVD. Mitochondrial dysfunction and cellular stress promote a pseudo-Warburg effect, shifting cells from efficient oxidative phosphorylation to anaerobic glycolysis and impairing homeostasis. Emerging therapies targeting hepatic metabolism to reduce cardiovascular risk and MASLD burden hold promise for future dual treatments.

**Summary:**

MASLD and ASCVD arise from common metabolic derangements that converge on shared cellular and molecular pathways. Defining these cross-tissue mechanisms may enable the development of integrated therapeutic approaches aimed at jointly mitigating hepatic and vascular injury, thus redefining treatment paradigms in cardiometabolic disease.

## Introduction: Pathogenesis of ASCVD and MASLD

 The leading cause of death globally for the past century is cardiovascular disease (CVD) [1]. Atherosclerotic cardiovascular disease (ASCVD), the buildup of fibro-fatty plaques in the vessel wall at locations of turbulent blood flow, underlie almost all CVDs, the most deadly being ischemic heart disease and stroke [1, 2]. Various comorbidities, termed ‘cardiometabolic diseases’, are growing in prevalence alongside ASCVD, the most alarming being metabolic dysfunction-associated steatotic liver disease (MASLD), currently affecting 38% of the population and growing rapidly [3, 4]. As the body’s central regulator of nutrient synthesis, storage, and catabolism, the liver is essential for maintaining metabolic homeostasis [5]. Yet, under chronic nutrient overconsumption, this regulatory hub becomes perturbed, fueling the progression of MASLD and ASCVD [5]. By regulating systemic lipid metabolism via secretion of very-low-density lipoproteins (VLDL) and controlling vascular inflammation through the release of systemic inflammatory mediators, metabolic dysregulation in the liver drives both ASCVD and MASLD concurrently [6, 7].

**Fig. 1:**
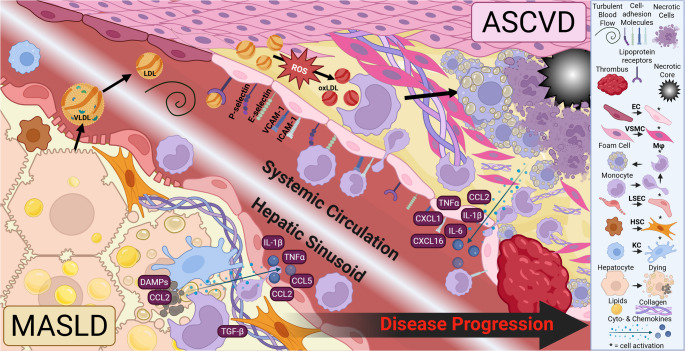
**The Complex Cellular Interplay during MASLD and ASCVD Progression**. During MASLD, hepatocytes synthesize, store and secrete lipids to maintain homeostasis. Lipid overabundance eventually overrides the normal mechanisms of lipid droplet formation leading to lipotoxic intermediates and hepatocellular damage. Liver sinusoidal endothelial cells (**LSECs**), which regulate substance exchange with the hepatocytes and systemic circulation via the liver sinusoids, lose their fenestrations from lipotoxic insults in a process called capillarization. Hepatocellular damage induces the release of damage-associate molecular patterns (DAMPs) and C-C motif chemokine ligand-2 (CCL2) which activate (denoted above as *) resident liver macrophages, or Kupffer cells ( **KCs**), inducing chemoattractant migration and the release of more inflammatory ‘danger signals’ like interleukin-1β (IL-1β), tumor necrosis factor-α (TNFα), CCL2, and CCL5 into circulation which recruit circulating monocytes to infiltrate the liver and become macrophages. Infiltrating macrophages secrete transforming growth factor-β (TGF-β) which, along with the inflammatory chemokines and cytokines already present, activate nearby hepatic stellate cells (**HSCs**) which synthesize and secrete collagen in a wound-healing response, promoting fibrosis. Simultaneously, during atherosclerosis progression, lipid-rich apoB-containing lipoproteins secreted from hepatocytes as very-low density lipoprotein (VLDL) break down into smaller low-density lipoprotein (LDL) in the bloodstream and are deposited within the subendothelial space at atheroprone regions characterized by turbulent blood flow, like arterial curvatures and branch points. Fluid shear stress at these sites activates endothelial cells (**ECs**), promoting the expression of lipoprotein receptors such as cluster of differentiation 36 (CD36), as well as the expression of cell adhesion molecules like P-selectin, E-selectin, vascular and intracellular cell adhesion molecules (VCAM-1 and ICAM-1 respectively), which promote the infiltration of circulating immune cells. Cytokines and chemokines including TNF-α, IL-1β, IL-6, CCL2, C-X-C motif ligand 1 (CXCL1), and CXCL16 released from ECs attract circulating monocytes to transmigrate into the subendothelial space and differentiate into macrophages. These professional phagocytes begin to take up LDL and oxidized-LDL (oxLDL) particles; the latter are generated through modification by reactive oxygen species (ROS). OxLDL uptake triggers macrophages to become lipid-overabundant and more quiescent, metabolically hindered ‘foam cells’, which accumulate to form atheromas. Inflammation, lipotoxicity, and hypoxia, promote foam cell necrosis, which is further exacerbated by impaired efferocytosis, or clearing of apoptotic cells, by macrophages. Necrotic cellular debris accumulate, creating ‘necrotic cores’. Simultaneously, the medial layer of vascular smooth muscle cells (**vSMCs**) becomes activated, shifting into a less contractile but more proliferative and migratory phenotype, surrounding the necrotic core and secreting collagen. This leads to the formation of a fibrous cap, which stabilizes the lesion. Macrophages degrade the fibrous cap by secreting matrix-metalloproteinases (MMPs) thinning it, eventually promoting plaque rupture and thrombus formation, leading to a cardiovascular event

MASLD is characterized by hepatic steatosis, the accumulation of lipid droplets in over 5% of hepatocytes [8]. Arguably the most metabolically active cells in the body, hepatocytes maintain systemic lipid homeostasis. Increased intracellular free fatty acids (FFAs) due to enhanced uptake and biosynthesis are stored as triacylglycerols (TAGs) within lipid droplets, a compensatory mechanism which sequesters toxic lipids, and in the case of overabundance leads to cellular damage (Fig. 1) [9, 10]. Liver sinusoidal endothelial cells (LSECs), interfacing blood and liver, mediate proper nutrient exchange through their fenestrations, which are lost during MASLD (LSEC capillarization) [11]. Lipotoxicity and cellular damage induces the release of inflammatory mediators from hepatocytes, which activate resident liver macrophages, or Kupffer cells (KCs) to secrete inflammatory cytokines into circulation [12]. In result, circulating monocytes are recruited into the liver, differentiate into macrophages, and phagocytose injured cells in an attempt at restoring homeostasis [12]. Simultaneously, combined secretion of factors from hepatocytes, KCs, and infiltrating macrophages trigger the activation of quiescent hepatic stellate cells (HSCs), which secrete extracellular matrix (ECM) components around injured sites generating fibrosis [13]. Through the complex interplay of these cells (Fig. 1), unchecked MASLD worsens, leading to a more advanced disease characterized by hepatocellular ballooning and inflammation termed metabolic dysfunction-associated steatohepatitis (MASH) [8]. Although MASH can be reversed with timely intervention, it often results in irreversible outcomes, including cirrhosis and hepatocellular carcinoma (HCC), which significantly increase mortality risk [8, 14]. In fact, MASH is rapidly overcoming all other causes of HCC, the third-leading cancer related mortality worldwide [14]. Despite the plethora of liver-related complications resulting from MASLD, the leading cause of death in patients with MASH is CVD [3, 5].

ASCVD is initiated when excess lipid-rich VLDL particles are secreted from hepatocytes and broken down into LDL, and deposited within the arterial subendothelial space, particularly at sites of disturbed flow [1, 15]. In these ‘athero-prone’ regions, oscillatory fluid shear stress activates endothelial cells (ECs, Fig. 1), promoting the expression of receptors which uptake these lipoproteins, alongside cell adhesion molecules which facilitate interactions with circulating immune cells [15]. Enhanced subendothelial lipid deposition induces the release of inflammatory and monocyte-attracting factors driving monocyte differentiation into macrophages [15, 16]. Once within the vessel wall, these professional phagocytes begin clearing and metabolizing LDL particles [15]. Though initially beneficial, macrophage uptake of modified LDL particles triggers a positive-feedback loop of unrestricted lipid uptake generating ‘foam cells’; unfunctional quiescent macrophages with impaired phagocytic capability [15, 16]. The plaque microenvironment promotes foam cell necroptosis and enhanced monocyte infiltration [15, 16]. Cellular debris accumulate within the plaque due to ineffective clearance, or efferocytosis, by foamy macrophages, creating ‘necrotic cores’ [15, 16], which promote plaque instability [17]. Simultaneously, vascular smooth muscle cells (vSMCs) become activated, undergo phenotypic switching, and migrate around the lipid-rich lesion, secreting collagen to stabilize the lesion and prevent rupture in a structure termed the ‘fibrous-cap’ [17]. Macrophages degrade this fibrous cap by secreting ECM-degrading enzymes [17]. Larger necrotic cores and thinner fibrous caps are features of rupture-prone atheromas, rendering patients susceptible to a potentially deadly manifestation of myocardial infarction or stroke [1].

MASLD and ASCVD are cardiometabolic diseases, often concurrent yet underdiagnosed, with overlapping altered metabolic pathways [5, 7]. While several therapeutics exist for ASCVD [18], and newly emerging therapeutics have recently been approved for MASLD/MASH [19], many patients experience recurrent cardiovascular events even with optimal pharmacologic targeting [20] and few therapeutics are effective for late-stage MASH-fibrosis [19]. Understanding the dysregulated metabolic pathways shared between these exceedingly comorbid diseases is necessary to develop direly needed therapeutics which can overcome the threats MASLD and ASCVD pose globally [21]. This review aims to shed light on the shared metabolic dysregulation in both MASLD and ASCVD and highlight areas lacking substantial investigation which warrant future research.

## Shared Dysregulated Metabolic Pathways in MASLD and ASCVD

**Fig. 2:**
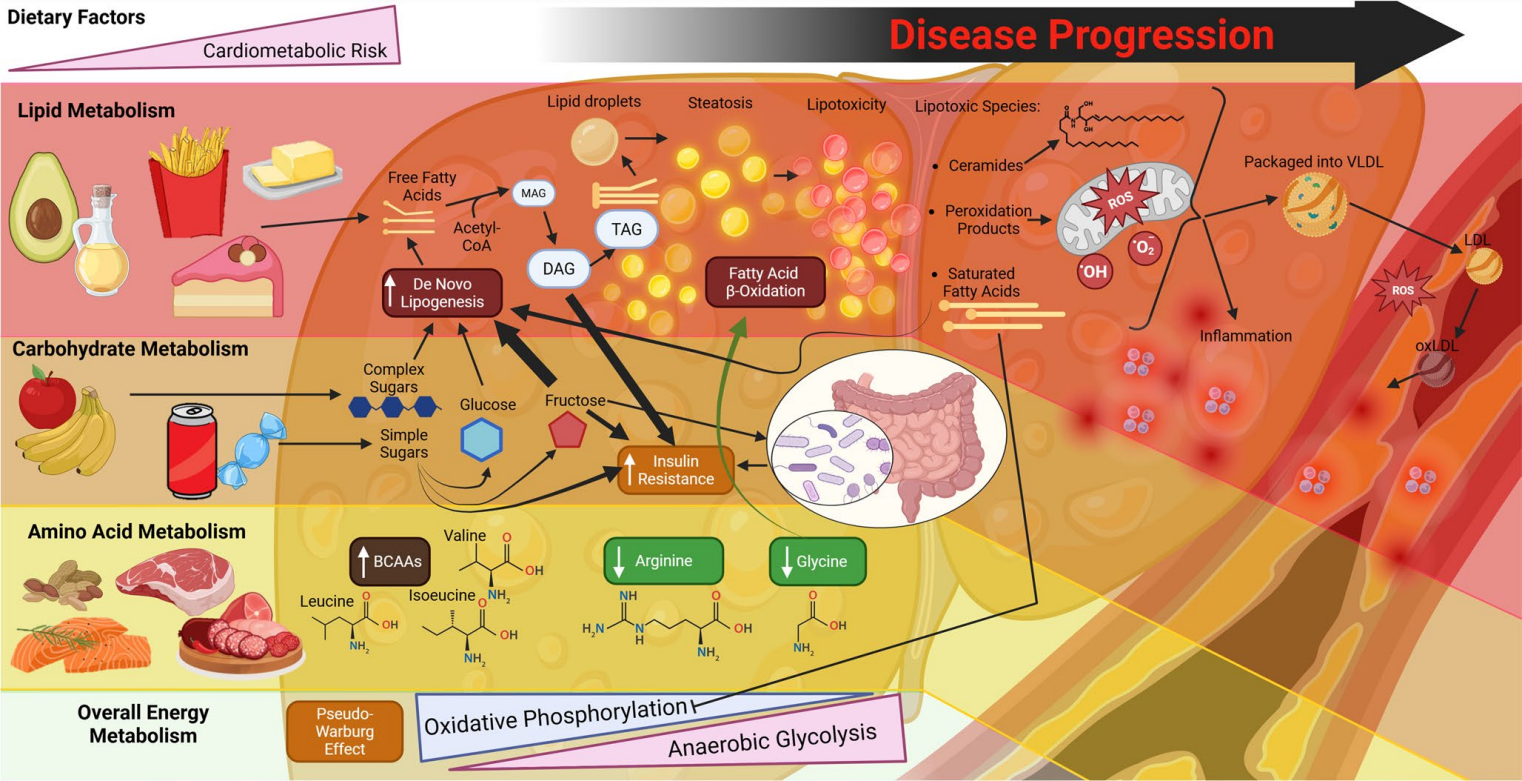
**The Dysregulated Metabolic Pathways Overarching MASLD and ASCVD**. Broadly, major avenues of metabolic regulation can be broken down into lipid, carbohydrate, amino acid, and overall energy metabolism, most of which is primarily orchestrated by the liver with cardiovascular consequences. Dietary intake of excess fatty acids and carbohydrates, drives enhanced lipid droplet formation and de novo lipogenesis, resulting in steatosis. Lipid overabundance overrides the mechanisms of lipid droplet formation, leading to the accumulation of lipotoxic species like diacylglycerols (DAGs), saturated fatty acids, lipid peroxidation products, and ceramides, which combined not only promote mitochondrial damage, reducing fatty acid oxidation capacity and promoting reactive oxygen species (ROS) generation and inflammation locally, but also systemically when these species are packed into VLDL and sent into systemic circulation, where they contribute to inflammation and oxidative stress within the plaque microenvironment. Simple sugars, especially fructose, trigger de novo lipogenesis and insulin resistance, which is partially mediated by gut microbiota. DAGs and fructose promote insulin resistance, which perpetuates hyperglycemia. Amino acid metabolism, affected by the quantity and quality of dietary protein, is widely altered in MASLD and ASCVD, with branched-chain amino acids (BCAAS) typically increased with disease severity, while amino acids like glycine and arginine are decreased with disease severity. The variety of gluco-lipotoxic insults during MASLD and ASCVD trigger and overall shifts from oxidative phosphorylation to anaerobic glycolysis, a process deemed the ‘Pseudo-Warburg Effect’. These interconnected pathways concurrently participate in the MASH-ASCVD cardiometabolic disease

It is widely accepted that the major dysregulated metabolic pathway in MASLD and ASCVD is lipid metabolism [1, 9, 10]. Later, the importance of carbohydrates [22], and more recently, amino acid metabolism [6, 17, 23–30] in both MASLD and ASCVD came into view. Through understanding the overarching metabolic pathways which contribute to MASLD and ASCVD pathogenesis (Fig. 2), we can begin to appreciate various therapeutic approaches to combat cardiometabolic disease. Here, we overview the metabolic disturbances in MASLD and ASCVD beginning with lipids and key lipotoxic species, followed by carbohydrate, amino acid, and overall energy metabolism, to illustrate the shared dysregulation of these diseases.

Lipid homeostasis at both the systemic and organ level requires an intricate balance of uptake, biosynthesis, degradation, and clearance [9, 31]. An overreliance on any of these pillar processes will collapse balanced lipid metabolism into an avalanche of metabolic dysfunction. In MASLD, hepatic steatosis is driven by excessive uptake of fatty acids, which overwhelm the livers’ metabolic capacity, resulting in accumulation of toxic intermediates that promote cellular stress and injury [9]. Enhanced *de novo* lipogenesis (DNL) [31], a characterizing feature of MASLD, promotes ASCVD through enhanced lipoprotein secretion alongside circulating FFAs [32]. These FFAs, both in the bloodstream and directly from the digestive tract post-prandially, are cleared by the liver through the hepatic portal vein and first-pass metabolism. While storing FFAs in lipid droplets is a protective compensatory response initially [9, 31], overabundance of FFAs, alongside diminished fatty acid β-oxidation (FAO) [9], eventually impair lipid droplet formation and FFA handling, leading to lipotoxicity [9, 10, 24, 31, 33].

Some lipid species with substantial evidence as drivers of both MASH and atherosclerosis include saturated fatty acids (SFA), diacylglycerol (DAG), ceramides, and lipid peroxidation products [10, 33]. SFAs contribute to MASLD and ASCVD by inducing mitochondrial damage, oxidative stress, inflammatory signaling pathways, and ultimately lipoapoptosis in hepatocytes, ECs, and macrophages [11, 15, 33]. In addition to excessive dietary intake, a significant portion of SFAs are derived from DNL triggered by excessive carbohydrate consumption [10, 33]. SFAs can be esterified to create monoacylglycerol (MAG), DAG, or triacylglycerol (TAG) [9]. Impaired lipid droplet formation leads to surplus DAGs, which block insulin signaling, promoting systemic insulin resistance [9, 10], a common feature of cardiometabolic disease which also drives DNL [34].

Other lipid species contributing to insulin resistance are ceramides, which are formed via conjugation of fatty acids to a sphingosine backbone [35]. These lipotoxic species are abundant in the plasma and liver of both MASH and ASCVD patients, and drive insulin resistance, inflammation, and mitochondrial dysfunction [35, 36]. Recent lipidomic studies uncovered that specific ceramides, varying by chain length and saturation, determine lipotoxicity in MASLD and atherosclerosis. This is supported by the emerging clinical risk scores, CERT1 and CERT2, which predict patient mortality based on ceramide species [35]. Another family of lipotoxic species are lipid peroxidation products, which promote inflammation and disease pathogenesis in both MASH and atherosclerosis by acting as signaling molecules, driving cellular damage, inflammation, and immune invasion [1, 33]. Mitochondrial damage render various metabolic processes dysfunctional in MASLD and ASCVD, dramatically increasing reactive oxygen species (ROS) in both the plaque and hepatic tissues, consequently generating lipid peroxides [37]. In atherosclerosis, oxidized LDL (oxLDL) particles alter cellular metabolism not only to drive macrophage foam cell formation, but also in smooth muscle cells, arguably the majority of foamy plaque cells [38], while oxidized phospholipids promote oxidative stress, inflammation, and thrombus formation [1, 39].

While dyslipidemia drives MASLD and ASCVD, increased carbohydrate intake aggravates dyslipidemia by promoting DNL and insulin resistance [33, 34]. Prolonged hyperglycemia promotes both enhanced insulin secretion and insulin resistance, together stimulating hepatic hormone sensitive lipase which breaks down TAGs stored in adipose tissue, further increasing circulating FFAs [34]. Simple sugars, fructose especially, induce insulin resistance and DNL, driving MASLD and ASCVD pathogenesis, directly increasing hepatic DAG and TAGs [33, 40]. Recently, the importance of gut carbohydrate metabolism in cardiometabolic diseases has gained increasing attention, as specific species can promote either insulin resistance or insulin-sensitivity [41]. Furthermore, microbial amino acid metabolism further impacts systemic host energy-balance [26, 42], aligning with the altered amino acid metabolism driving both MASLD and ASCVD [17, 23–30].

Higher levels of most circulating amino acids are associated with increased MASLD and ASCVD severity [17, 24, 26, 27, 30]. Accordingly, amino acids have recently been uncovered as a crucial carbon source for DNL [43]. Some amino acids are protective in cardiometabolic diseases, however, with recent studies uncovering the importance of glycine in regulating redox balance and promoting FAO in both hepatocytes and macrophages during MASLD and atherosclerosis [23, 26, 28, 30, 44, 45], while arginine-derived polyamines crucially regulate macrophage efferocytosis [46, 47], posing amino acid metabolism as valuable therapeutic avenue, worthy of detailed exploration. Branched-chain amino acids (BCAAs) have a highly controversial role in cardiometabolic diseases; while consistently increased in the plasma of patients with insulin resistance, MASLD, and atherosclerosis [24, 43], supplementation with BCAAs ameliorates a variety of liver diseases [48, 49], and the actions of individual metabolites on particular cell types seems highly specific [50]. Further, the cell-specific context of amino acid metabolism also impacts whether it is protective or detrimental to metabolic disease pathogenesis. For example, while leucine supplementation prevents foam-cell formation [23, 27, 51], leucine also promotes mechanistic target of rapamycin (mTOR) activation in macrophages to drive atherosclerosis through impaired autophagy and mitophagy [50].

Generating cellular energy, predominantly adenosine triphosphate (ATP), can either occur through the slower oxygen-dependent and highly efficient process of mitochondrial oxidative phosphorylation, or the faster cytoplasmic anaerobic breakdown of glucose, or anaerobic glycolysis [52]. While anaerobic glycolysis is an inefficient means of generating energy, it is significantly faster than oxidative phosphorylation (10–100 times), underpinning a defining metabolic shift in tumor cells deemed the Warburg effect [52]. Given the role of mitochondrial dysfunction as a driver of the Warburg effect in cancer, and its contribution to both MASH and ASCVD pathogenesis [10], emerging research suggests a ‘pseudo-Warburg effect’ that may underlie cardiometabolic diseases [53]. The shift from oxidative phosphorylation to anaerobic glycolysis has been suggested as a key mechanism driving MASH to HCC transition [54], though further research is necessary to elucidate these mechanisms and their therapeutic implications.

The complex interplay of these dysregulated metabolic pathways driving both MASLD and ASCVD (Fig. 2) is further exacerbated by the variety of specific cell types playing major roles in disease pathogenesis. Therefore, we elaborate on the most recent understandings of dysregulated metabolism in key cell types crucial to both ASCVD and MASLD development, and some of the intricate crosstalk between them.

## Dysregulated Cellular Metabolic Pathways Linking MASLD and ASCVD

### Liver Cells

#### Hepatocytes

Arguably the most metabolically active cells, hepatocytes orchestrate systemic lipid, carbohydrate, and amino acid metabolism [55]. In MASLD, increased FFA uptake, enhanced DNL, as well as impaired FAO and lipid droplet formation, together induce lipotoxicity and mitochondrial dysfunction [10]. While carbohydrates promote DNL, recently, amino acids have been identified as a major carbon source for proper DNL in hepatocytes [43]. Accordingly, the amino acids serine and glycine, which are integral to one-carbon metabolism (1CM) encompassing a variety of reactions which transfer one-carbon units [56], are widely dysregulated in cardiometabolic diseases. Interestingly, circulating glycine and the glycine: serine ratio are significantly reduced in MASLD/MASH and ASCVD patients [24, 27, 57, 58], while glycine and glycine-based treatments protect against both diseases in preclinical animal models [27, 44, 45, 58]. Glycine depletion is dictated by the reverse activity of serine hydroxymethyltransferase 2 (SHMT2) in the liver [56], which has been shown to exacerbate acetaminophen-induced hepatotoxicity in MASLD through impaired glutathione synthesis [28]. Impaired glycine formation due to suppressed hepatic alanine-glyoxylate aminotransferase (AGXT) activity in both MASH and ASCVD, is linked to the overproduction of oxalate [26, 29, 58, 59]. Hepatocellular oxalate accumulation has recently been uncovered as a central component of MASH pathogenesis by inhibiting FAO [29], while in atherosclerosis, oxalate accumulation in macrophages promotes inflammation and mitochondrial dysfunction [58]. Together, these recent discoveries emphasize the importance of dysregulated hepatocyte amino acid metabolism in MASH and ASCVD as a potential driver and therapeutic target.

At the junction of amino acid and lipid metabolism, emerging studies highlight the importance of hepatocyte ceramide metabolism in both MASH and ASCVD progression. Ceramides can either be derived from *de novo* biosynthesis, through the rate-limiting conjugation of serine and palmitoyl-CoA to produce sphinganine [60], or from the metabolism of downstream sphingomyelin species by hydrolases [61]. Hepatic sphinganine was recently discovered as a predictor of disease severity across a variety of murine models of concurrent MASH and ASCVD [6]. Furthermore, sphinganine increases lipid accumulation and inflammation in both hepatocytes and macrophages [6]. Recently, the importance of sphingomyelin hydrolysis in generating hepatic ceramides was uncovered. In MASH, lipotoxicity induces a sirtuin 1 (SIRT1)-dependent upregulation of sphingomyelin phosphodiesterase 3 (SMPD3), which emerges as a major source of lipotoxic ceramides [61]. These ceramides are released in extracellular vesicles and promote the activation of HSCs and KCs [61]. Recent reports have further uncovered that increased ceramides secreted from hepatocytes within VLDL drive trans-fat induced ASCVD [60], posing ceramide metabolism as an important link between MASH and atherosclerosis.

#### Liver Sinusoidal Endothelial Cells

After hepatocytes, LSECs represent the most abundant cell population in the liver [62], reflecting the organ’s extensive vascularization. LSECs form a permeable barrier between the blood and hepatocytes, characterized by fenestrations that enable macromolecule exchange, lack of a basement membrane allowing direct hepatocyte contact, and high endocytic capacity for lipoprotein clearance [11]. Disruption of these functions also contributes to dyslipidemia by altering lipid permeability and fostering ASCVD development [11]. Under physiological conditions, LSECs rely on glucose-derived pyruvate, short- and medium-chain fatty acids and glutamine to fuel oxidative phosphorylation while largely avoiding β-oxidation of long-chain fatty acids, reflecting a metabolically plastic EC phenotype adapted to the liver environment [63]. In early MASLD, ECs develop transient mitochondrial dysfunction compensated by enhanced glycolysis, promoting a pro-inflammatory phenotype even before morphological capillarization occurs [64]. In more aggressive MASH models, LSEC capillarization forms a continuous basement membrane, impairing lipid transfer before KC activation [65]. As fibrosis progresses, increased matrix stiffness recruits glycolytic enzymes to focal adhesions, amplifying C-X-C motif ligand 1 (CXCL1)-mediated inflammatory signaling and perpetuating vascular dysfunction [66]. Collectively, the metabolic shift of LSECs from oxidative phosphorylation to glycolysis not only drives hepatic inflammation and fibrogenesis, but also accelerates MASH progression.

#### Kupffer Cells

Liver macrophages are central to the development of MASLD, where they orchestrate inflammatory signaling, influence fibrosis progression, and contribute to tissue repair and resolution [67]. In healthy liver tissue, the majority of macrophages are embryonically derived KCs localized in hepatic sinusoids [12, 68]. KCs act as professional phagocytes, removing pathogens alongside apoptotic and necrotic debris [68]. As MASLD advances, the KC population declines due to increased cell death, which is compensated by the infiltration of circulating monocytes that differentiate into monocyte-derived macrophages (MdMs) within the liver [12]. Although KC depletion contributes to this cellular turnover, monocyte recruitment can occur independently [69], and the mechanisms driving KC loss are still poorly understood. This highlights the need to explore molecular pathways regulating KC homeostasis and the maintenance of hepatic immune-metabolic balance.

KCs express G protein-coupled receptors (GPRs), that act as metabolic sensors for circulating metabolites to maintain homeostasis [70]. GPR3 recently gained attention as an important regulator of metabolic reprogramming in KCs. Activation of GPR3 initiates a rapid shift from oxidative phosphorylation to glycolysis by promoting the assembly of β-arrestin-2 with key glycolytic enzymes, thereby enhancing their enzymatic activity [70]. Surprisingly, despite this glycolytic shift which is typically associated with a pro-inflammatory phenotype, GPR3 activation supports an anti-inflammatory phenotype and protects against MASLD progression [70]. This positions GPR3 as a central mediator of KC metabolic remodeling and a therapeutic target for steatotic liver diseases. Recent single-cell transcriptomics have delineated two major KC subsets in MASLD: a dominant KC1 population (85%) and a smaller KC2 subset (15%), each exhibiting distinct transcriptional and proteomic signatures (Table 1) [68]. KC2 cells exhibit a lipid-handling metabolic profile driven by elevated cluster of differentiation 36 (CD36) expression, whereas KC1 cells maintain an immune dominant program with minimal metabolic specialization, aligning them more closely with classical antigen-presenting KCs [68]. However, the precise identity of KC2 remains elusive, owing to their notable transcriptional similarity to LSECs [68]. Future research on KC metabolism, depletion, and monocyte recruitment, will offer critical insight into MASLD pathogenesis and unveil potential immunometabolic targets for therapeutic intervention.

**Table 1:** Monocyte subsets in MASLD & ASCVD- emerging genomic and proteomic signatures from scRNA seq

Population	Gene/Protein expressed	Phenotype	Species	Ref
**Resident Hepatic Mφs**				
Resident KCs (Conserved)*	*CD5L*,* VSIG4*,* SLC1A3*,* CD163*,* FOLR2*,* TIMD4*,* MARCO*,* GFRA2*,* ADRB1*,* TREM2 SLC40A1*,* HMOX1*, *SLC16A9*, and *VCAM1*	Professional phagocytes	Mouse and Human	[71]
KC1 (85%)	CD206^lo^ESAM^−^	Antigen presentation, immune surveillance	Mouse	[68]
KC 2 (15%)	CD206^hi^ESAM^+^	Lipid handling	Mouse	[68]
**Monocyte Derived Hepatic Mφs (MdMs)**				
Ly6C^hi^ Mφs*	*Ly6C* ^hi,^ *Chil3*^hi^, *Ccr2*^hi^, *Lyz2* ^hi^, *Fn1*^hi^, and *Trem2*^−^	Inflammatory and fibrotic	Mouse	[72]
Monocyte-derived Kupffer cells (MoKCs),	*Adgre1*,* Vsig4*,* Clec4f*, and *Clec1b; Timd4*^−^, *Gpnmb*^inter^ *Cd9*^inter^, and *Trem2*^hi^	Inflammatory and fibrotic	Mouse	[72]
Lipid-associated Mφs (LAMs)	*Trem2*^hi^, *Spp1*^hi^, *Cd9*^hi^, *Fapb5*^hi^, and *Ccr2*^hi^	Inflammation resolution	Mouse	[72]
**Atherosclerotic Plaque Mφs**				
Resident Mφs	*Lyve1*,* Cx3cr1*,* Folr2*,* Cd206*,* F13a1*,* Ccr2*,* Sepp1*,* Pf4*,* Gas6*,* Cd206*,* Timd4*, and *Ccl8*	Adventitia - Phagocytosis	Mouse	[73]
Proliferating Mφs	*Birc5*,* Mki67*,* Ube2c*,* Cenpf*,* Top2a*,* Prc1*, and *H1f6*	Intima/adventitia - Proliferation	Mouse	[73]
Inflammatory Mφs	*Nfkbia*,* Tnf*,* Nlrp3*,* IL-1β*,* Cxcl1*, and *Cxcl2*	Intima - Inflammatory response and antigen presentation	Mouse	[73]
Interferon-inducible Mφs	*Irf7*,* Ifit3*,* Isg15*,* and Ifit1*	Intima - Unknown	Mouse	[73]
Foamy/ Trem2^+^ Mφs	*Trem2*,* Gpnmb*,* Spp1*,* Fabp4*, and *Fabp5*	Lipid-core edge in the intima - Lipid consumption and phagocytosis	Mouse	[73]

*These resident KC markers are conserved across 7 species, including mice and humans [71]. Mφ; Macrophage. Gene and protein modifiers: ^lo^low expression; ^inter^intermediate expression; ^hi^high expression; ^+^presence; ^−^absence. Gene and protein acronyms: *Adgre1*Adhesion G protein-coupled receptor E1, *ADRB1 *adrenoceptor beta 1, *Birc5 *baculoviral IAP repeat containing 5 (survivin), *Ccl8 *C-C motif chemokine ligand 8, *Ccr2 *C-C motif chemokine receptor 2, *CD163 *CD163 molecule (hemoglobin scavenger receptor), *CD206 *(Mrc1) mannose receptor C-type 1, *CD5L *CD5 molecule-like (apoptosis inhibitor of macrophage), *CD9* cluster of differentiation 9 (CD9) molecule (Tetraspanin-29 29), *Cenpf *centromere protein F, *Chil3 *chitinase-like 3, *Clec1b *C-type lectin domain family 1 member B, *Clec4f *C-type lectin domain family 4 member F, *Cx3cr1 *C-X3-C motif chemokine receptor 1, *Cxcl1 *C-X-C motif chemokine ligand 1, *Cxcl2 *C-X-C motif chemokine ligand 2, *ESAM *endothelial cell adhesion molecule, *F13a1 *coagulation factor XIII A chain, *Fabp4 *fatty acid-binding protein 4, *Fabp5 *fatty acid-binding protein 5, *Fn1 *fibronectin 1, *FOLR2 *folate receptor beta, *Gas6 *growth arrest-specific 6, *GFRA2 *GDNF family receptor alpha 2, *Gpnmb *glycoprotein nonmetastatic melanoma protein B, *H1f6 *H1.6 linker histone, *HMOX1 *heme oxygenase 1, *Ifit1 *interferon-induced protein with tetratricopeptide repeats 1, *Ifit3 *interferon-induced protein with tetratricopeptide repeats 3, *Il1b *interleukin 1 beta, *Irf7 *interferon regulatory factor 7, *Isg15* interferon stimulated gene 15, *Ly6c* lymphocyte antigen 6 complex, locus C1, *Lyve1* lymphatic vessel endothelial hyaluronan receptor 1, *Lyz2* lysozyme C-2, *MARCO* macrophage receptor with collagenous structure, *Mki67* marker of proliferation Ki-67, *Nfkbia* NFKB inhibitor alpha, *Nlrp3* NLR family pyrin domain containing 3, *Pf4* platelet factor 4, *Prc1* protein regulator of cytokinesis 1, *Sepp1* selenoprotein P, *SLC16A9* solute carrier family 16 member 9, *SLC1A3* solute carrier family 1 member 3, *SLC40A1* solute carrier family 40 member 1 (Ferroportin), *Spp1* secreted phosphoprotein 1 (osteopontin), *TIMD4* T-cell immunoglobulin and mucin domain containing 4, *Tnf* tumor necrosis factor, *Top2a* topoisomerase (DNA) II alpha, *TREM2* triggering receptor expressed on myeloid cells 2, *Ube2c* ubiquitin-conjugating enzyme E2C, *VCAM1* vascular cell adhesion molecule 1, *VSIG4* V-set and immunoglobulin domain-containing 4.

#### Hepatic Infiltrating Monocytes

Monocyte infiltration is a major driver of MASLD/MASH progression [12]. Once recruited to the liver, inflammatory monocytes differentiate into macrophages, whose fate and function are shaped by hepatic microenvironmental cues [12]. These macrophages adopt a distinct metabolic profile, generally subdivided into either pro-inflammatory macrophages, which exhibit increased glycolysis and TCA cycle activity, or regenerative/pro-resolving macrophages, which depend more on FAO and the arginase pathway [74]. One important TCA cycle aberration in macrophages involves immune-responsive gene-1 (IRG1), the enzyme responsible for converting the TCA intermediate cis-aconitate into the anti-inflammatory metabolite itaconate. In liver macrophages, β-arrestin-2 promotes IRG1 ubiquitination, lowering itaconate production and shifting TCA flux toward enhanced succinate dehydrogenase activity, thereby increasing mitochondrial ROS and driving pro-inflammatory polarization [75]. In MASH, β-arrestin-2 is markedly upregulated in both hepatic macrophages and circulating monocytes, further accelerating IRG1 degradation, depleting itaconate [75]. Surprisingly, in a recent multiomics analysis of concurrent MASLD and ASCVD murine models, hepatic itaconate emerged as one of the most consistently increased metabolites, posing itaconate metabolism as a valuable link between MASLD and ASCVD pathogenesis [6]. Whether inhibiting β-arrestin-2 or promoting IRG1 stability to increase itaconate can be pharmacologically targeted to ameliorate inflammation during the cardiometabolic disease remains to be uncovered [6, 75].

Beyond alterations in the TCA cycle, hepatic macrophages in MASLD/MASH also exhibit profound metabolic rewiring of gluconeogenic, lipid uptake, and FAO pathways, for example through the coordinated regulation of forkhead box protein O1(FOXO1)-driven gluconeogenesis and CD36-mediated lipid uptake, supporting efficient β-oxidation [76]. This coordination is disrupted in MASH, where the stress-induced activating transcription factor-3 (ATF3), a key stabilizer of macrophage glucolipid metabolism, is significantly downregulated [76]. ATF3 suppresses FOXO1-driven gluconeogenesis and promotes CD36-dependent fatty acid uptake, thus inducing FAO [76]. Restoring ATF3 function in hepatic macrophages therefore offers an avenue for MASH intervention [76]. A similar metabolic imbalance occurs with the E2F transcription factor 2 (E2F2), which is markedly reduced in MASH-associated macrophages, and depletion of which exacerbates hepatic steatosis, inflammation, and HSC activation by driving leucine–mTORC1–dependent metabolic reprogramming towards increased glycolysis and impaired mitochondrial function [77]. Beyond the canonical pro-inflammatory and pro-resolving macrophage phenotypes, more macrophage niches have recently been identified creating a mosaic of specific subtypes and functions (Table 1) [68, 71, 72]. Given their distinct identities, the metabolic dysregulation in these macrophage subsets remains poorly understood and warrants further investigation. Luckily, the rapid progress currently underway through single-cell transcriptomics [71, 72], alongside emerging techniques like spatially-resolved mass spectrometry imaging [78], will allow dissection of dysregulated metabolism in specific immune-cell subsets which drive cardiometabolic dysregulation to help guide rational therapeutic design.

#### Hepatic Stellate Cells

In their homeostatic state, HSCs reside quiescent in the space of Disse and function as the body’s primary retinol reserve sequestered into intracellular lipid droplets [79]. However, in response to liver injury, HSCs are activated (aHSCs), acquiring a myofibroblast-like, contractile phenotype [79]. aHSCs contribute to tissue repair by producing ECM components, but excessive ECM deposition ultimately drives fibrosis and scarring, making HSC activation a key event in the progression of chronic liver diseases [79]. The activation switch to a myofibroblast-like phenotype imposes extensive energetic demands and requires substantial metabolic reprogramming [80].

Glycolysis is one of the earliest and strongest upregulated pathways driving HSC activation [80]. Upregulation of 6-phosphofructo-2-kinase/fructose-2,6-biphosphatase-3 (PFKFB3), the key regulator of glycolytic flux, is essential for this shift [80]. Disruption of factors that stabilize *PFKFB3* mRNA, such as the post-transcriptional regulator cytoplasmic polyadenylation element binding protein 4 (CPEB4), blocks HSC activation [80]. Along with increased energy production, glycolysis promotes pyruvate formation and channeling towards lactate, a driver of HSC activation [81]. Deletion of hexokinase-2, the first committed step of glycolysis, as well as pharmacological lactate lowering via inhibition of lactate dehydrogenase (LDH), therefore showed attenuated levels of aHSCs [81]. Another glycolytic checkpoint is pyruvate kinase M2 (PKM2) which depending on its isoform, promotes HSC activation (dimeric) or prevents nuclear localization and suppresses fibrogenesis (tetrameric) [82].

Lipid and amino acid metabolism also play vital roles in regulating HSC phenotype. Inhibition of acetyl-CoA carboxylase, the rate-limiting step of DNL, not only decreased aHSCs, but also induced fibrosis regression due to lower fatty acid accumulation and lipotoxicity [83]. Glutamine also serves as an alternative energy source via glutaminolysis [84]. Alanine-serine-cysteine transporter-2, a high-affinity glutamine transporter, is elevated in aHSCs and its inhibition induces senescence [84]. Downstream, glutamate dehydrogenase links amino acid metabolism to the TCA cycle, and inhibitors of this enzyme, such as, epigallocatechin gallate or SIRT4 reduce metabolic flux into the TCA cycle, suppressing HSC activation [85]. Given their importance in hepatic fibrosis, pharmacologically targeting intrinsic metabolic pathways central to HSC activation could ultimately prevent or reverse fibrotic liver diseases.

### Plaque Cells

#### Endothelial Cells

ECs form the inner lining of blood vessels and are in constant contact with circulating blood, making them the first responders to hemodynamic and metabolic stressors that drive atherosclerosis [1]. Their metabolism determines vascular homeostasis, influencing lipid uptake, inflammatory signaling, and crosstalk with vascular and immune cells [86]. Despite their direct access to oxygen, ECs rely predominantly on anaerobic glycolysis for ATP production under physiological conditions, thereby conserving oxygen for underlying smooth muscle and parenchymal tissues [86]. In regions of disturbed laminar flow, this glycolytic dependence is exacerbated by hypoxia-inducible factor-1α (HIF-1α) activation, promoting endothelial activation and NADPH oxidase 4 (NOX4)-mediated ROS production, contributing to vascular inflammation via enhanced interleukin-8 (*IL8*), C-C motif chemokine ligand-2 (*CCL2*), and vascular cell adhesion molecule 1 (*VCAM1*) expression [87].

The metabolic fate of fatty acids fulfills crucial metabolic and redox-regulatory functions in ECs. In quiescent ECs, FAO is upregulated, not to support energy production, but to sustain TCA cycle activity and preserve redox homeostasis through NADPH regeneration [86]. Concurrently, acetyl-CoA derived from FAO serves as an essential substrate for post-translational protein acetylation, preventing endothelial-to-mesenchymal transition (EndMT) by stabilizing SMAD7-mediated signaling [88]. In contrast, aberrant acetylation of the mitochondrial chaperone cyclophilin D alters mitochondrial permeability transition, promoting oxidative stress, endothelial dysfunction and hypertension [89]. Further, dysregulated metabolism of polyunsaturated fatty acids in atheroprone ECs through upregulation of soluble epoxide hydrolase promotes mitochondrial dysfunction and ROS generation [90].

The metabolism of ECs relies heavily on nitrogen-rich amino acids, like glutamine, which maintains TCA cycle activity and contributes to mitochondrial function [86]. Arginine is used for NO synthesis, through which ECs regulate vascular tone and communicate with their environment [86]. This process, catalyzed by endothelial nitric oxide synthase (eNOS), requires sufficient levels of arginine and a balanced redox state to maintain enzymatic coupling and sustain NO production [86]. Disruption of glycolysis or depletion of its co-factors results in eNOS uncoupling, where eNOS shifts from producing NO to generating superoxide anions, lowering NO bioavailability and amplifying oxidative stress [86]. This imbalance drives endothelial activation, leukocyte adhesion, and vascular inflammation, collectively promoting atherosclerosis [86]. Overall, the balance between glycolysis, FAO, and amino acid-driven redox pathways dictates whether ECs remain quiescent or shift toward a dysfunctional, pro-inflammatory state under hemodynamic or metabolic stress [86, 88, 90]. Disruptions in these circuits converge on excess ROS generation, impaired NO signaling, and activation of pro-atherogenic programs.

#### Vascular Infiltrating Monocytes and Macrophages

During early atherosclerosis, circulating monocytes infiltrate the vessel wall and differentiate into macrophages [1, 15]. Plaque macrophages exhibit remarkable metabolic plasticity, adapting their energy pathways in response to environmental cues, in turn influencing their polarization and impacting plaque initiation, progression, and regression (Table 1) [73]. In early lesions, oxLDL taken up by macrophages promotes a pro-inflammatory phenotype by driving a metabolic shift away from FAO toward aerobic glycolysis [73]. Enhanced glycolysis, disrupted TCA cycle flux, and accumulation of intermediates such as succinate and citrate, stabilize HIF-1α and amplify inflammatory gene expression [73, 91]. Additionally, hypoxia, ROS, and inflammatory stimuli, concurrently activate HIF-1α, dramatically inducing anaerobic glycolysis [73, 91]. Dysregulated amino acids, particularly leucine, additionally exacerbate this glycolytic transition through activating the mTOR-HIF-1α-glycolysis axis, potentially through leucine intermediate acetyl-CoA production [50]. Collectively, enhanced anaerobic glycolysis in macrophages increases ROS production, expression of pro-inflammatory mediators, and accelerates plaque development [91].

HIF-1α has also been reported to participate in inflammation resolution in macrophage efferocytosis, a crucial step in atherosclerosis regression [46, 47, 92]. This pro-resolving phenotype is also dictated by amino acid metabolism. For example, the arginine- and ornithine-derived metabolites, putrescine and spermidine, maintain expression of the efferocytosis receptor MER proto-oncogene, tyrosine kinase (MerTK) and enhance IL-10 production, thereby activating adenosine monophosphate-activated protein kinase (AMPK) and HIF-1α via mitochondrial superoxide production, promoting plaque regression [46, 47, 92]. However, if arginine is metabolized by inducible nitric oxide synthase (iNOS) to generate NO, a subsequent pro-inflammatory response occurs [47], highlighting the intricate balance of amino acid metabolism in dictating macrophage fate during atherosclerosis. This is further exemplified by the importance of glycine in preventing macrophage foam cell formation by suppressing VLDL uptake [23], underscoring the importance of amino acid metabolism in macrophage phenotype.

Additionally, macrophage lipid metabolism is central to atherosclerosis progression. Uptake of oxLDL via scavenger receptors leads to the accumulation of both free and esterified cholesterol, overwhelming cholesterol efflux and transport mechanisms, driving foam cell formation [15, 16]. Cholesterol overload triggers mitochondrial dysfunction, ROS generation, and ultimately promotes necrotic core expansion and plaque instability [15, 16]. Foam cell formation is further exacerbated by elevated ceramide levels, both in circulation and in plaques, which disrupt actin polymerization, impairing LDL degradation [93]. Resulting cholesterol overload induces cytotoxicity and oxidative stress, suppressing FAO and impairing mitochondrial oxidative phosphorylation further [15, 16]. While dysregulated metabolism drives the inflammatory phenotype of macrophages, our current understanding of immunometabolism is just scratching the surface of the intricacies at play, and how to strategically use this knowledge to mitigate plaque progression.

#### Vascular Smooth Muscle Cells

vSMCs constitute the predominant cells in the vessel wall. Their role in atherosclerosis is pivotal, as transitions between contractile, synthetic, and inflammatory phenotypes critically influence plaque formation, stability, and vascular remodeling [17]. Under physiological conditions, mechanical stress and vasoactive signals maintain the contractile phenotype via glucose-driven oxidative phosphorylation which sustains energy production and calcium handling [17]. Upon platelet-derived growth factor stimulation, vSMCs transition to a synthetic phenotype, where dimerization of PKM2 redirects pyruvate toward anaerobic glycolysis, promoting proliferation and migration in an extracellular signal-related kinase (ERK)-mTOR-signal transducer and activator of transcription 3 (STAT3)-dependent manner [94]. When anaerobic glycolysis persists under exposure to pro-inflammatory cytokines, oxidized lipoproteins, and cholesterol loading, vSMCs adopt an inflammatory phenotype that exhibits macrophage-like features but impaired lipid handling [38]. vSMC-derived foam cells display reduced expression of cholesterol efflux transporters, ATP-binding cassette transporters A1 and G1, leading to inefficient cholesterol clearance, cholesterol accumulation, and sustained inflammation destabilizing the plaque [38].

Between the contractile and synthetic states lies the fibro-myocyte phenotype, which co-expresses contractile and ECM genes and contributes to fibrous cap stability [95]. Spatial metabolomic profiling of human atherosclerotic plaques reveals localized pyruvate enrichment within stable fibrous caps [78], consistent with enhanced glycolytic flux supporting ECM synthesis by fibro-myocytes [80]. Another phenotypic switch into the ‘osteogenic-like vSMCs’, driven by oxidative stress and phosphate overload, promotes vascular calcification through runt-related transcription factor 2-mediated transcriptional reprogramming [96]. Simultaneously, senescent vSMCs accumulate with aging and metabolic stress, lose their proliferative capacity, and adopt a pro-inflammatory secretory phenotype that amplifies endothelial dysfunction and plaque instability [97]. This phenotypic heterogeneity reflects a continuum of metabolic rewiring linking vascular remodeling in ASCVD with systemic metabolic stress common in MASH.

## Immuno-Metabolic & Fibro-Metabolic Responses in ASCVD and MASLD

In MASH, infiltrating macrophages secrete inflammatory and profibrogenic mediators that activate HSCs stimulating fibrosis and contribute to a systemic inflammatory burden that elevates cardiovascular risk [12, 13]. In atherosclerosis, vSMCs determine plaque stability, however, MMPs from infiltrating monocytes and cytotoxic signals from foam cells render the fibrous cap thin and rupture-prone [17]. Furthermore, vSMCs adopt different phenotypes contributing to plaque progression, including foam cell [38] and osteoblast-like-vSMC, contributing to plaque calcification [96]. Interestingly, exogenous lactate promotes vSMC calcification [98]. Taken together with the role of lactate as a driver of HSC activation in MASLD [81], its accumulation in activated macrophages [99], and its enrichment within the necrotic core [78], these findings paint a compelling picture of how anaerobic glycolytic flux toward lactate production feeds fibroblast-macrophage crosstalk to worsen cardiometabolic disease. This is further supported by the recent discovery of lactate as one of the most consistently upregulated hepatic metabolites in concurrent MASH and atherosclerosis, which drives hepatocyte lipotoxicity and inflammation in macrophages [6]. How to target this axis, to mitigate fibrosis in MASH without thinning the fibrous cap in atherosclerosis, while promoting the ‘resolving-macrophage’ phenotype, remains to be discovered.

Systemic inflammation underlies the clinical interrelationship between MASLD and ASCVD [6], substantiated by epidemiological and meta-analytic studies demonstrating a higher incidence of cardiovascular events and mortality among individuals with MASLD [5]. Systemic inflammatory markers elevated in MASLD, including CCL2, CCL5, C-reactive protein and IL-6 [6, 7, 29], show strong associations with endothelial dysfunction and subclinical atherosclerosis, supporting the concept that hepatic inflammation amplifies vascular risk. Furthermore, metabolomic analyses reveal shared alterations in lipid species and amino acid-derived metabolites among individuals with MASLD and those with ASCVD, highlighting convergent metabolic disturbances that may propagate inflammation across both hepatic and vascular tissues [6, 7, 17, 23–30]. This ‘immuno/fibro-metabolic’ response is a shared hallmark of MASH and ASCVD, the extent of which indicates disease outcomes, yet few existing treatments successfully address this crucial component of concurrent pathology [19, 100, 101].

## Pharmacologic Interplay Between MASH and ASCVD Therapies

The close pathophysiologic relationship between MASH and ASCVD extends to pharmacologic management. Here, we briefly overview how ASCVD therapies affect hepatic lipid metabolism, inflammation, and fibrogenesis, then discuss emerging MASH drugs that may favorably alter cardiovascular risk profiles. This section summarizes the current evidence on shared mechanisms, overlapping benefits, and areas requiring further investigation.

Lipid-lowering strategies remain central to ASCVD treatment. Statins, the most widely prescribed agents in atherosclerosis, directly inhibit HMG-CoA reductase preventing hepatic cholesterol synthesis [102]. Historically, statin use in MASH was approached with caution due to concerns regarding hepatotoxicity [102]. However, recent evidence challenges this limitation, as a large meta-analysis found no association between statin therapy and worsening liver enzymes in patients with MASLD [102], and one study reported significant reductions in ALT levels of MASH patients [103]. Ezetimibe, which reduces intestinal cholesterol absorption, has shown mixed effects in MASLD. While treatment is associated with decreased hepatic fibrosis, it also increased hepatic long-chain fatty acid accumulation, likely due to impaired FAO and altered glucose metabolism, though larger clinical trials are necessary to draw stronger conclusions [104]. Mounting evidence positions proprotein convertase subtilisin/kexin type 9 (PCSK9) inhibition as the most potent lipid lowering therapy [105], supported by a phase 3 clinical trial of an oral PCSK9 inhibitor showing promising lipid and safety profiles [106]. Individuals with PCSK9 loss-of-function mutations exhibit protection against hepatic steatosis, immune infiltration, and fibrosis [107]. Similarly, PCSK9 inhibition in murine models recapitulates these findings, alongside downregulated DNL and upregulated lipolysis/FAO pathways [108]. Together, these findings underscore the bidirectional benefits of lipid-lowering therapy, suggesting that pharmacologic modulation of cholesterol metabolism may simultaneously mitigate atherogenesis and hepatic lipotoxicity in patients with overlapping diseases.

The use of antithrombotic therapy in ASCVD is generally reserved for secondary prevention [109]. While acetylsalicylic acid (aspirin) does not confer additional benefits beyond statin therapy in preventing MASH progression in humans [110], dual antiplatelet therapy with clopidogrel or ticagrelor prevented downregulation of genes involved in FAO, lipolysis and cholesterol metabolism in mice with MASH-derived HCC [111]. Lastly, although no studies have directly assessed the effects of warfarin or acenocoumarol in MASH, emerging evidence indicates that vitamin K exerts protective effects, attenuating steatohepatitis and fibrosis in both mice and humans [112]. These findings warrant further investigation into the impact of anticoagulant therapies on hepatic metabolism and disease progression.

Although hypertension is a well-stablished risk factor for ASCVD and associated with MASLD in observational studies [113], the effects of antihypertensive drugs on hepatic metabolism appear to be modest. Activation of HSCs is accompanied by local angiotensin II synthesis [13] and the use angiotensin-converting enzyme inhibitors has been associated with lower risk of MASH derived cirrhosis and HCC [114]. In the same study, angiotensin receptor blockers failed to prevent those outcomes; however, more recent evidence suggests that losartan may act as effective adjuvant to anti PD-1 immunotherapies in MASH-derived HCC [115]. Lastly, amlodipine, a calcium channel blocker, has been shown to alleviate MASLD progression through modulation of gut microbiome [116].

The use of specific antihyperglycemic agents in patients with type 2 diabetes is recommended to reduce ASCVD risk [109]. Sodium-glucose cotransporter-2 inhibitors particularly have shown additional hepatometabolic benefits in a clinical trial, associated with a higher likelihood of MASLD regression and a lower incidence of adverse liver-related outcomes compared with other antidiabetic drugs [117]. Since that report, the therapeutic landscape for ASCVD management has been profoundly transformed by glucagon-like peptide-1 (GLP-1) receptor agonists with demonstrated profound cardiovascular benefits [118]. Those benefits extend further, as semaglutide has now been approved for the treatment of MASH [119]. The first agent specifically developed to target the underlying metabolic and fibrotic mechanisms of MASH, resmetirom, was recently approved [100]. This selective thyroid hormone receptor–β agonist enhances hepatic FAO and mitochondrial function, reduces intrahepatic fat and fibrosis, and simultaneously improves the systemic lipid profile by lowering LDL-cholesterol and triglycerides [100]. Although these findings suggest potential cardiovascular benefits, the direct impact of resmetirom on ASCVD risk remains to be established in long-term outcome studies.

Peroxisome proliferator-activated receptor (PPAR) agonists represent a promising class of agents capable of modulating both hepatic and vascular pathology. PPARα activation promotes FAO and improves plasma lipid profiles, PPARγ regulates lipid storage and insulin sensitivity, and PPARδ promotes FAO and energy expenditure. Pemafibrate, a selective PPARα modulator, lowers triglycerides and remnant lipoproteins while improving hepatic enzyme levels, although its long-term cardiovascular outcomes remain under investigation [120]. Elafibranor, a dual PPARα/δ agonist, showed beneficial metabolic and hepatic effects in early studies but failed to meet histologic endpoints in phase 3 trials (NCT02704403). Lanifibranor, a pan-PPAR agonist, has demonstrated significant histologic improvement in MASH, with reductions in steatosis and lobular inflammation without worsening of fibrosis [101] and is currently in phase 3 clinical trials (NCT04849728). Collectively, these results highlight the therapeutic promise and complexity of PPAR targeting, and its potential to simultaneously mitigate ASCVD and MASH.

## Conclusions

Taken together, dysregulated metabolism across a plethora of cell-types dramatically influences the shared pathogenesis of MASLD and ASCVD. Understanding the temporal metabolic shifts that contribute to cell fate and function, alongside the intricate crosstalk at play, will surely lay the foundation for groundbreaking therapeutics to overcome these epidemics. We have highlighted the convergence of metabolic and cardiovascular pharmacology, suggesting that agents initially designed for ASCVD prevention may also modulate MASH progression through shared molecular pathways. Therapeutics such as GLP-1 receptor agonists, though initially designed for treating diabetes, were later used for obesity, and are now showing promise in treating MASH and ASCVD, supporting the notion that overall metabolic syndrome drives these diseases. Understanding these cross-disease effects could guide the rational design of therapies that simultaneously target hepatic and vascular injury, ultimately reshaping the clinical management of ASCVD and MASH.

## Key References

- Das S, Anand SK, McKinney MP, et al. Sex-based multiomics analysis uncovers metabolic and molecular mediators linking MASH and atherosclerosis. JHEP Reports. Dec 2 2025. 10.1016/j.jhepr.2025.101703.
  - ○ This study integrates multiomic analyses of diet-induced MASH and atherosclerosis in mouse models combined with human data from MASH patients to identify conserved metabolic features that illuminate pathological crosstalk and provide a translational platform for therapeutic target discovery and novel drug development.
- Ghrayeb A, Finney AC, Agranovich B, et al. Serine synthesis via reversed SHMT2 activity drives glycine depletion and acetaminophen hepatotoxicity in MASLD. Cell Metab. Jan 2 2024;36(1):116-129.e7. 10.1016/j.cmet.2023.12.013
  - ○ This paper is the first to describe reverse activity of serine hydroxymethyltransferase 2 in the liver and its role in driving glycine depletion during the early stages of MASLD.
- Das S, Finney AC, Anand SK, et al. Inhibition of hepatic oxalate overproduction ameliorates metabolic dysfunction-associated steatohepatitis. Nat Metab. Oct 2024;6(10):1939-1962. 10.1038/s42255-024-01134-4
  - ○ This study identifies oxalate as a key driver of MASH progression, disrupting FAO and promoting inflammation through monocyte recruitment, while simultaneously opening new avenues for pharmacological intervention. Notably, oxalate has also been shown to promote atherosclerosis, exemplifying a mechanistic link between the two diseases discussed in this review.
- Jiang J, Gao Y, Wang J, et al. Hepatic sphingomyelin phosphodiesterase 3 promotes steatohepatitis by disrupting membrane sphingolipid metabolism. Cell Metab. May 6 2025;37(5):1119-1136.e13. 10.1016/j.cmet.2025.01.016
  - ○ This article unveils how sphingomyelin hydrolysis at the cell membrane by SMPD3 elevates ceramide levels in MASH and regulates lipid uptake and extracellular vesicle release, mediating activation of HSCs and KCs.
- Hu S, Li R, Gong D, et al. Atf3-mediated metabolic reprogramming in hepatic macrophage orchestrates metabolic dysfunction-associated steatohepatitis. Sci Adv. Jul 26 2024;10(30):eado3141. 10.1126/sciadv.ado3141
  - ○ This research article reports that ATF3 expression is reduced in CD68⁺ liver macrophages in patients with MASH, and identifies ATF3 as a central regulator of macrophage metabolic reprogramming. The authors demonstrate that ATF3 controls the glucose-fatty acid cycle and that its loss exacerbates hepatic steatosis, inflammation, and fibrosis.
- Rho H, Terry AR, Chronis C, Hay N. Hexokinase 2-mediated gene expression via histone lactylation is required for hepatic stellate cell activation and liver fibrosis. Cell Metab. Aug 8 2023;35(8):1406-1423.e8.
  - ○ This article highlights the role of lactate in activating hepatic stellate cells via histone lactylation and proposes pharmacological targeting of HK2 as a potential therapeutic strategy for liver fibrosis
- Dikalova A, Fehrenbach D, Mayorov V, et al. Mitochondrial CypD Acetylation Promotes Endothelial Dysfunction and Hypertension. Circ Res. May 24 2024;134(11):1451-1464. 10.1161/circresaha.123.323596
  - ○ This study reveals that CypD acetylation in endothelial cells promotes hypertension by driving oxidative stress, metabolic dysfunction, and endothelial damage; correcting this acetylation restores vascular function and lowers blood pressure.
- Harrison SA, Bedossa P, Guy CD, Schattenberg JM, Loomba R, Taub R, et al. A Phase 3, Randomized, Controlled Trial of Resmetirom in NASH with Liver Fibrosis. N Engl J Med. 2024;390(6):497-509. 10.1056/NEJMoa2309000
  - ○ This phase 3 clinical trial in adults with biopsy-confirmed MASH showed that 52 weeks of treatment led to MASH resolution without worsening of fibrosis and improved fibrosis by at least one stage without deterioration in the NAFLD activity score. Importantly, these data supported the approval of the first drug indicated for MASH.
- Ballantyne CM, Gellis L, Tardif JC, Banka P, Navar AM, Asprusten EA, et al. Efficacy and Safety of Oral PCSK9 Inhibitor Enlicitide in Adults With Heterozygous Familial Hypercholesterolemia: A Randomized Clinical Trial. JAMA. 2025. 10.1001/jama.2025.20620.
  - ○ This phase 3 randomized clinical trial of an oral PCSK9 inhibitor enlicitide demonstrated a sustained ~60% LDL cholesterol reduction, overcoming the major limitation of injectable administration. Beyond lipid lowering, oral PCSK9 inhibition may confer dual benefits by modulating hepatic lipid metabolism and inflammation, bridging therapeutic strategies for MASH and ASCVD.
- Sanyal AJ, Newsome PN, Kliers I, Østergaard LH, Long MT, Kjær MS, et al. Phase 3 Trial of Semaglutide in Metabolic Dysfunction-Associated Steatohepatitis. N Engl J Med. 2025;392(21):2089-99. 10.1056/NEJMoa2413258.
  - ○ This phase 3 trial demonstrated significant improvements in steatohepatitis and fibrosis among patients with MASH, leading to FDA approval of semaglutide for this indication. These results establish GLP-1 receptor agonists as a cornerstone therapy linking metabolic, hepatic, and cardiovascular disease management.

## Data Availability

No datasets were generated or analysed during the current study.
